# Requirement of the LtsA Protein for Formation of the Mycolic Acid-Containing Layer on the Cell Surface of *Corynebacterium glutamicum*

**DOI:** 10.3390/microorganisms9020409

**Published:** 2021-02-16

**Authors:** Yutaro Kumagai, Takashi Hirasawa, Masaaki Wachi

**Affiliations:** 1 School of Life Science and Technology, Tokyo Institute of Technology, Yokohama 226-8501, Japan; yutaro.kumagai@aist.go.jp (Y.K.); thirasawa@bio.titech.ac.jp (T.H.); 2 Cellular and Molecular Biotechnology Research Institute, National Institute of Advanced Industrial Science and Technology, Tsukuba 305-8565, Japan

**Keywords:** *Corynebacterium glutamicum*, LtsA, cell-surface structure, mycolic acid

## Abstract

The *ltsA* gene of *Corynebacterium glutamicum* encodes a *purF*-type glutamine-dependent amidotransferase, and mutations in this gene result in increased susceptibility to lysozyme. Recently, it was shown that the LtsA protein catalyzes the amidation of diaminopimelate residues in the lipid intermediates of peptidoglycan biosynthesis. In this study, intracellular localization of wild-type and mutant LtsA proteins fused with green fluorescent protein (GFP) was investigated. The GFP-fused wild-type LtsA protein showed a peripheral localization pattern characteristic of membrane-associated proteins. The GFP-fusions with a mutation in the N-terminal domain of LtsA, which is necessary for the glutamine amido transfer reaction, exhibited a similar localization to the wild type, whereas those with a mutation or a truncation in the C-terminal domain, which is not conserved among the *purF*-type glutamine-dependent amidotransferases, did not. These results suggest that the C-terminal domain is required for peripheral localization. Differential staining of cell wall structures with fluorescent dyes revealed that formation of the mycolic acid-containing layer at the cell division planes was affected in the *ltsA* mutant cells. This was also confirmed by observation that bulge formation was induced at the cell division planes in the *ltsA* mutant cells upon lysozyme treatment. These results suggest that the LtsA protein function is required for the formation of a mycolic acid-containing layer at the cell division planes and that this impairment results in increased susceptibility to lysozyme.

## 1. Introduction

A non-pathogenic species of coryneform bacteria, *Corynebacterium glutamicum*, has been known as a glutamate-overproducing microorganism [1,2] and utilized for the production of various amino acids, such as lysine [3,4], valine [5] and threonine [6]. *C. glutamicum* can also be used as a host for the production of useful chemicals, including organic acids and alcohols [7,8]. Moreover, *C. glutamicum* is a Gram-positive bacterium with high G+C content phylogenetically related to mycobacteria and related taxa, known as Corynebacterineae.

Glutamate production by *C. glutamicum* is triggered by the limitation of biotin, which is essential for its growth, and addition of Tween 40 or penicillin [9,10,11]. Biotin is a cofactor for acetyl-CoA carboxylase, which is responsible for fatty acid biosynthesis. Tween 40 also affects fatty acid biosynthesis. Penicillin inhibits the biosynthesis of peptidoglycan, which is a component of the bacterial cell wall. These inducing treatments for glutamate production affect cell surface formation in *C. glutamicum*. Furthermore, the mechanosensitive channel, NCgl1221, is responsible for the secretion of glutamate produced in *C. glutamicum* [12]. During glutamate production by *C. glutamicum*, it is thought that the NCgl1221 protein senses the change in tension of the cell surface and the resultant conformational change within the NCgl1221 protein occurs in response to these inducing treatments. As a result, glutamate is secreted through this mechanosensitive channel to the outside of the cells [13,14,15,16,17]. Therefore, analysis of the relationship between cell surface formation and glutamate production is important for understanding the mechanism underlying glutamate production by *C. glutamicum*.

*C. glutamicum* has thick cell walls consisting of an inner peptidoglycan layer, which invaginates to form the septum, and the outer mycolic acid-containing layer, which functions as a permeability barrier and renders cells highly tolerant to antibiotics and host defense mechanisms [18,19]. The peptidoglycan in *C. glutamicum* is formed by glycan chains consisting of *N*-acetylglucosamine and *N*-acetylmuramic acid cross-linked with the pentapeptides. However, *C. glutamicum* exhibits tolerance to the lytic enzyme lysozyme, which catalyzes hydrolysis of the glycan chain in peptidoglycan, based on the existence of a mycolic acid-containing layer in the cell wall.

In order to understand cell surface structure formation in *C. glutamicum*, we have been analyzing lysozyme-sensitive mutants of it. In our previous report, we identified *ltsA,* which is responsible for lysozyme sensitivity and temperature-sensitive growth of the lysozyme-sensitive mutant strain, KY9714 [20]. It was shown that *ltsA* mutants produced glutamic acid without the inducing treatments [21]. The *ltsA* gene encodes a *purF*-type glutamine-dependent aminotransferase, which shows high homology with glutamine-dependent asparagine synthetases from various organisms. However, the *ltsA* gene could not complement the asparagine auxotrophy of the *Escherichia coli asnA asnB* mutant. It is known that the N-terminal glutamine amido transfer (GAT) domain in the *purF*-type glutamine-dependent amidotransferases is required for their amido transfer reactions [22]. The N-terminal region of the *C. glutamicum* LtsA protein exhibited high homology with the GAT domain of the *purF*-type glutamine-dependent amidotransferases. In particular, the second cysteine residue, which is necessary for the amido transfer reaction of the *purF*-type glutamine-dependent amidotransferases [22,23,24], is conserved in the *C. glutamicum* LtsA protein. Moreover, it was reported that the LtsA protein catalyzes the amidation of diaminopimelate (DAP) residues in the lipid intermediates of peptidoglycan biosynthesis [25].

In this study, in order to investigate the roles of LtsA protein in cell surface formation of *C. glutamicum*, we examined the intracellular localization of fusion proteins featuring wild-type and mutant LtsA proteins with a green fluorescent protein (GFP) in *C. glutamicum* cells. Moreover, formation of the peptidoglycan layer and mycolic acid-containing layer was analyzed by selective staining of these cell-surface structures whose methods were described in our previous report [26]. Our results indicate that the LtsA protein localized at the cell division site is involved in mycolic acid-containing layer formation. Moreover, we also found that the C-terminal domain of the LtsA protein is required for its peripheral localization in *C. glutamicum* cells.

## 2. Materials and Methods

### 2.1. Bacterial Strains, Media and Growth Conditions

*C. glutamicum* wild-type strain KY9611 and the *ltsA* mutant strains, KY9714, KY9704, KY9706 and KY11939 [20,21], were used in this study. For plasmid construction, *E. coli* JM109 (*recA1 endA1 gyrA96 thi hsdR17* (r_k_^–^ m_k_^+^) *e14*^–^ (*mcrA*^–^) *supE44 relA1* Δ(*lac-proAB*)/F’(*traD36 proAB*^+^ *lacI*^q^ *lacZ*Δ*M15*)) was employed. For transformation of *C. glutamicum*, the plasmids extracted from the transformants of *E. coli* JM110 (*dam dcm supE44 hsdR17 thi leu rpsL1 lacY galK galT ara tonA thr tsx* Δ(*lac-proAB*)/F(*traD36 proAB*^+^ *lacI*^q^ *lacZ*Δ*M15*)) were utilized. For the overproduction of 6 × histidine (His_6_)-tagged LtsA protein, *E. coli* BL21(DE3) (F^–^ *ompT hsdS* (r_B_^–^ m_B_^–^) *gal dcm* λ(DE3)) (Novagen, Madison, WI, USA) was used as a host.

Both *C. glutamicum* and *E. coli* were cultivated at 30 °C or 37 °C in L medium consisting of 1% peptone, 0.5% yeast extract, 0.5% NaCl and 0.1% glucose (pH 7.0 adjusted with NaOH). To prepare L plates, 1.5% agar was added to L medium. If required, 10 µg mL^–1^ or 20 µg mL^–1^ of kanamycin for *C. glutamicum* and *E. coli*, respectively, and 50 µg mL^–1^ lysozyme was added to the L medium.

### 2.2. Analysis of Temperature- and Lysozyme-Sensitivity in C. glutamicum

To check temperature sensitivity, serially 10^–1^ to 10^–5^-fold diluted overnight cultures of *C. glutamicum* were spotted on L plates and the plates were then incubated at 30 °C or 37 °C for 1 d. As for the verification of lysozyme sensitivity, serially diluted cultures were spotted onto L plates containing 50 µg mL ^–1^ of lysozyme and then the plates were incubated at 30 °C for 1 d.

### 2.3. Construction of GFP-Fused ltsA Genes and Microscopic Observation of Intracellular Localization of LtsA-GFP Fusion

The DNA fragments for wild-type *ltsA* and its *ltsA9706* and *ltsA11939* alleles were amplified by polymerase chain reaction (PCR) from the chromosomes of the KY9611, KY9706 and KY11939 strains, respectively, using LA PCR kit v.2.1 (Takara Bio Inc., Shiga, Japan) and the set of primers, 5′-ACGCATGCTAGCCTCAGGTGGAATTCCTAA-3′ and 5′-GGGTTTAGGCTTTAAGCTAGCAAGCTCGAG-3′. The amplified fragments were cloned into *the Nhe*I site of pQBI T7-GFP (Qbiogene, Inc., Carlsbad, CA) to obtain the inframe *gfp*-fused *ltsA* wild-type and mutant genes. The *ltsA*-*gfp* fusions were cloned into the *Eco*RI site of *E. coli*–*C. glutamicum* shuttle vector pHT1 [27] and the resulting plasmids were introduced into the KY9714 mutant to investigate intracellular localization of GFP-fused mutant LtsA proteins. The direction of fusion genes was designed so as to be opposite to that of transcription from the *lac* promoter on pHT1; the *ltsA-gfp* fusions were transcribed from the native *ltsA* promoter.

In addition, the GFP-fusion of the mutant LtsA truncated in the C-terminal region, which is the same as that in the *ltsA9704* mutant, was also constructed. The truncated *ltsA* gene, which is designated *ltsA*ΔC, was amplified by PCR from KY9611 chromosome DNA using the set of primers, 5′-ACGCATGCTAGCCTCAGGTGGAATTCCTAA-3′ and 5′-CCTTGACCAGGCTAGCGCCGCGCATCCAGG-3′. The amplified fragment was cloned into the *Nhe*I site of pQBI T7-GFP. The *ltsA*ΔC*-gfp* fusion was next cloned into the *Eco*RI site of pHT1, and the resulting plasmid was introduced into the KY9714 mutant.

Microscopic observation of intracellular localization of the GFP-fused mutant LtsA proteins was carried out using a fluorescent microscope (Axioskop 2, Carl Zeiss Co. Ltd., Oberkochen, Germany) equipped with a CCD camera (MicroMax, Roper Industries Inc., Bogart, GA, USA).

### 2.4. Construction of the Recombinant Strains Carrying Mutant ltsA Alleles

The DNA fragments carrying the mutation sites in the *ltsA9706* allele were amplified by PCR from the chromosomal DNA of the KY9706 mutant and the set of primers, 5′-TTTGAATTCAGGAGGATTTTTCATT-3′ and 5′-TCCAAGGTCGACATGATCTTAGGAA-3′. The amplified fragment was then cloned into the *Eco*RI-*Sal*I sites of pK18*mobsacB* [28]; the resulting plasmid was designated psacBltsA9706-5′. For *ltsA11939*, the set of primers, 5′-TTGACTCAACCGGAATTCCGCCGCT-3′ and 5′-CTCCTTCAACTAAAGAGGATCCGTT-3′, was used for amplification of the DNA fragment carrying the mutation in the *ltsA11939* allele from the chromosomal DNA of the KY11937 mutant. The obtained fragment was cloned into the *Eco*RI-*Bam*HI sites of pK18*mobsacB*; the resulting plasmid was designated psacBltsA11939-3′.

The psacBltsA9706-5′ and psacBltsA11939-3′ plasmids were introduced into the wild-type KY9611 strain, and kanamycin-resistant transformants were obtained. The introduced plasmid was integrated into the *ltsA* locus on the chromosome of the kanamycin-resistant transformants because the plasmids cannot replicate in *C. glutamicum* cells. Plasmid-integrated transformants exhibited sucrose sensitivity owing to the presence of the *sacB* gene on the plasmid. If the plasmid was looped out from the chromosome of the transformant by homologous recombination at the *ltsA* locus, it was expected that the resulting strains showed tolerance to sucrose and carried either wild-type or mutant *ltsA* genes. Therefore, overnight cultures of the transformants on L liquid medium without kanamycin addition were plated on L plates containing 20% sucrose, and the plates were incubated at 30 °C to obtain sucrose-tolerant strains. As it was expected that the strains carrying the *ltsA* mutant alleles showed sensitivities to both kanamycin and lysozymes, we examined the sensitivities of the sucrose-tolerant strains to kanamycin and lysozymes. Introduction of the *ltsA9706* and *ltsA11939* mutations was confirmed by colony PCR and nucleotide sequencing. The strains carrying the *ltsA9706* and *ltsA11939* alleles were designated YK06 and YK39, respectively.

### 2.5. Preparation of Polyclonal Rabbit Antiserum Containing Anti-LtsA Antibodies

For purification of the LtsA protein, the His_6_-tagged LtsA protein was obtained. The *ltsA* gene was amplified by PCR from KY9611 chromosome DNA using the set of primers, 5′-TTTGCGAATTCAGGCGCCTTTTATT-3′ and 5′-TAGGCTTTAAGACTTAAAGCTTGAC-3′), and cloned into the *Eco*RI-*Hin*dIII sites of pET-28b’, which was constructed by digestion with *Nco*I and *Bam*HI, blunting, and self-ligation of the expression vector, pET-28b(+) (Novagen). The resulting plasmid, pLtsA-His_6_, was introduced into the *E. coli* BL21(DE3). The transformants were cultivated in L medium and 2 µg mL^–1^ of IPTG to induce the expression of the *ltsA-his_6_* fusion gene at the early exponential phase. Four hours after IPTG addition, cells were harvested by centrifugation and suspended in 50 mM sodium phosphate buffer (pH 7.0). The harvested cells were disrupted by sonication and centrifuged again to remove cell debris. The supernatant was further ultracentrifuged at 100,000 × *g* at 4 °C, and the supernatant was then removed. The pellet containing the proteins associated with the cytoplasmic membrane was suspended in the sodium phosphate buffer and the proteins were solubilized with 2% Triton X-100. The LtsA-His_6_ protein in the solubilized protein extract was purified using a Histrap kit (Amersham Biosciences, Buckinghamshire, UK). The purified LtsA-His_6_ protein was injected into the rabbit, and serum containing anti-LtsA-His_6_ antibodies was obtained.

### 2.6. Observation of Intracellular Localization of LtsA by Indirect Immunofluorescence Hybridization

Indirect immunofluorescence hybridization was performed according to the methods reported by Hiraga et al. [29]. *C. glutamicum* cells at the exponential growth phase fixed in 90% methanol on ice for 5 min were spread on glass slides and then dried. After that, the cells on the slides were treated with 20 mg mL^–1^ lysozyme in 50 mM glucose and 25 mM Tris·HCl (pH 8.0) for 1 h and washed with phosphate-buffered saline containing 0.05% (*v*/*v*) Tween 20 (PBST) followed by treatment with methanol and acetone for 1 min each. For blocking, cells were treated with 2% (*w*/*v*) bovine serum albumin solution in PBST and then washed with PBST. Finally, the cells were incubated with rabbit antiserum including anti-LtsA antibodies as a primary antibody and Alexa Fluor 488-conjugated rabbit IgG (Molecular Probes, Eugene, OR) as a secondary antibody and observed by fluorescence microscopy.

### 2.7. Selective Staining of Peptidoglycan and Mycolic Acid-Containing Layers in C. glutamicum

As previously reported [26], we microscopically detected peptidoglycan and mycolic acid-containing layers in *C. glutamicum* by staining with fluorescent probes BODIPY FL vancomycin (Van-FL) (Molecular Probes) and rhodamine B-1,2-dihexadecanoyl-*sn*-glycero-3-phosphoethanolamine triethylammonium salt (DHPE) (Molecular Probes), respectively. Stock solutions of these fluorescent probes were prepared at 100 µg mL^–1^ of Van-FL in water and 10 µg mL^–1^ of DHPE in methanol. When staining the cells with Van-FL, a 1:1 mixture of Van-FL (100 µg mL^–1^) and vancomycin (100 µg mL^–1^) was used.

*C. glutamicum* cells during the exponential growth were fixed with 1.6% formaldehyde in phosphate-buffered saline (PBS) on ice for 1 h. After washing the fixed cells with PBS, the cells were spread on glass slides and then dried, followed by coating with poly-*L*-lysine. The fixed cells on glass slides were next stained with fluorescent probes for 15 min at room temperature and washed with saline followed by observation with fluorescence microscopy.

### 2.8. Microscopic Observation of Bulge Formation in C. glutamicum

*L* medium containing 20% sucrose, 50 µg mL^–1^ lysozyme and 2% low-melting agarose was layered on the 0.5% agarose on the slide glass and then solidified. Cells of the wild-type strain KY9611 and its *ltsA* mutant strain KY9714 grown at the mid-exponential phase, suspended in saline containing 20% sucrose, were spotted onto the agarose layer, and then cover glass was placed on the agarose layer. The prepared slides were incubated at room temperature and then cells on the slides were observed under a microscope in differential interference contrast (DIC) mode.

## 3. Results

### 3.1. Intracellular Localization of LtsA Protein in C. glutamicum Cells

**Figure 1:**
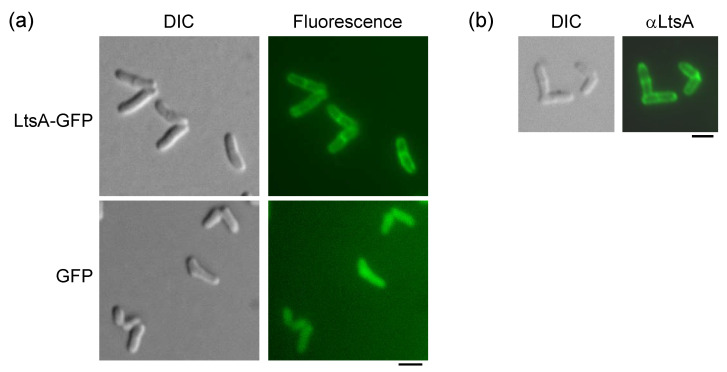
Intracellular localization of LtsA protein in *C. glutamicum* cells. (**a**) Fluorescent microscopic observation of *C. glutamicum* expressing *ltsA-gfp* fusion gene. Differential interference contrast (DIC) microscopy images and fluorescence microscopy images observing GFP or LtsA-GFP fusion (Fluorescence) in *C. glutamicum* cells are shown. For observation of localization of GFP and LtsA-GFP fusion, *C. glutamicum* KY9714 carrying plasmids with LtsA-GFP fusion (LtsA-GFP) and GFP alone (GFP) were used, respectively. A bar represents 2 μm. (**b**) Indirect immunofluorescence microscopic observation of intracellular localization of LtsA protein in *C. glutamicum*. DIC image and detection image of LtsA by anti LtsA antiserum (αLtsA) in the wild-type strain KY9611 are presented. A bar represents 2 μm.

To investigate the role of LtsA protein in the cell surface formation of *C. glutamicum*, we first assessed the intracellular localization of the LtsA protein in *C. glutamicum* cells by GFP-fusion analysis. The constructed *ltsA-gfp* fusion gene could complement the lysozyme sensitivity and temperature-sensitive growth of the *ltsA* mutant strain KY9714 (Supplementary Figure S1), indicating that the LtsA-GFP fusion protein is functional in *C. glutamicum* cells. The fusion gene was introduced into the KY9714 strain, and intracellular localization of LtsA-GFP fusion was observed through fluorescence microscopy. As shown in Figure 1a, peripheral localization of LtsA-GFP was observed, which is characteristic of membrane-associated proteins. In addition, fluorescence signals were observed at possible division sites at the center of the cells and the fluorescence intensity at the possible division sites was higher than that at the side wall.

To exclude the possibility of artificial localization owing to GFP fusion, we also observed the localization of LtsA in *C. glutamicum* cells by indirect immunofluorescence microscopy with an anti-LtsA antibody. As shown in Figure 1b, fluorescence images similar to those of LtsA-GFP-expressing cells were obtained, indicating that the LtsA protein indeed peripherally localized in *C. glutamicum* cells. It is notable that fluorescent signals were stronger at the possible division sites and cell poles than the side wall (Figure 1b). Similar tendency was also seen in the LtsA-GFP fusion expressing cells (Figure 1a). These results suggest that the LtsA protein is recruited to the division sites during cell division.

### 3.2. Requirement of C-Terminal Domain for Peripheral Localization of the LtsA Protein in C. glutamicum

As described in the Introduction, the LtsA protein has an N-terminal GAT domain, which is highly conserved among *purF*-type glutamine-dependent amidotransferases, but its C-terminal domain is not conserved and may be required for recognition of the amido acceptor in the lipid intermediate during peptidoglycan biosynthesis. In order to analyze how *ltsA* mutations affect the localization of the LtsA protein in *C. glutamicum* cells, we constructed recombinant strains expressing the mutant *ltsA* genes fused with *gfp* and observed intracellular localization of GFP-fused mutant LtsA proteins in *C. glutamicum* cells. The mutant alleles of *ltsA* used for construction of GFP fusions were *ltsA9706* carrying a missense mutation (substitution of the 80th glycine residue, which is highly conserved among *purF*-type amidotransferases [30], to aspartate; G80D) in the N-terminal GAT domain, *ltsA11939* carrying a missense mutation in the C-terminal domain (substitution of the 559th proline residue to serine; P559S), and *ltsA9704* carrying a nonsense mutation in the C-terminal domain (substitution of the codon for the 485th tryptophan residue to the opal stop codon; W485opal), which results in the formation of C-terminally truncated protein that we designated LtsAΔC. We constructed the *ltsA9706-gfp* and *ltsA11939-gfp* fusion genes, similar to wild-type *ltsA-gfp*. In the case of the *ltsA9704*, a fusion gene was designed so that the GFP was fused to the C-terminal end of the truncated LtsAΔC protein synthesized from the *ltsA9704* allele. These *gfp*-fused mutant *ltsA* genes were introduced into the *ltsA* mutant KY9714, and intracellular localization of the GFP-fused mutant LtsA proteins was observed.

**Figure 2:**
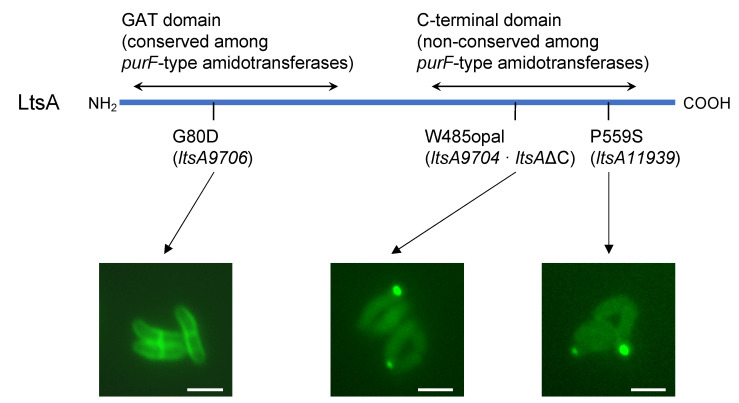
Intracellular localization of GFP-fusion of mutant LtsA proteins in *C. glutamicum* cells. In this figure, KY9714 cells expressing *ltsA9706-gfp*, *lts*AΔC*-gfp*, and *ltsA11939-gfp* gene fusions, respectively, are shown. Bars represent 2 μm.

As shown in Figure 2, the LtsA9706-GFP protein, which had a G80D substitution in the N-terminal domain, exhibited peripheral localization similar to that of wild-type LtsA-GFP. On the other hand, LtsA11939-GFP and LtsAΔC-GFP proteins, which carry the P559S substitution in the C-terminal domain and C-terminal truncation, respectively, did not show such peripheral localization, but dot-like fluorescence signals were observed in the cells (Figure 2). These results indicate that the C-terminal region of the LtsA protein is required for peripheral localization in *C. glutamicum* cells and that glutamine-dependent amidotransferase activity itself is not necessary for such localization.

### 3.3. Defect in Mycolic Acid-Containing Layer Formation in the C. glutamicum ltsA Mutant

**Figure 3:**
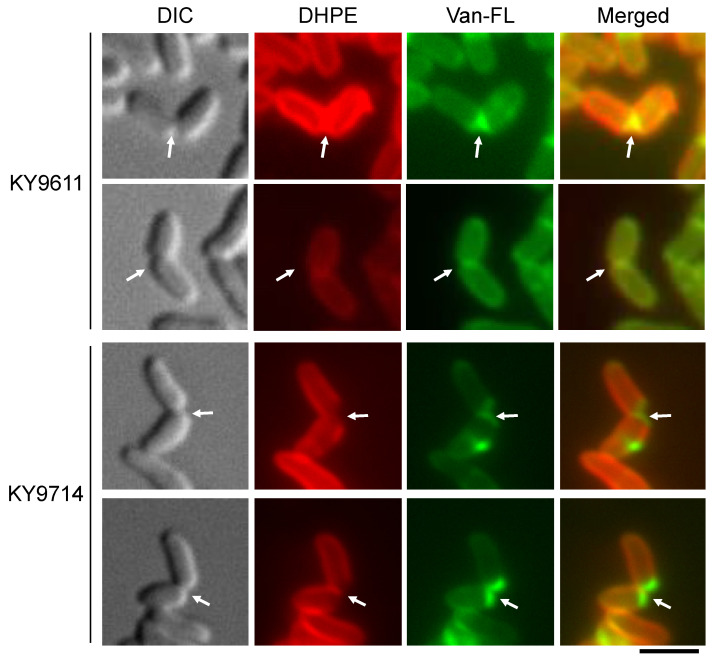
Microscopic observation of mycolic acid-containing layers at cell division sites in the *C. glutamicum* wild-type strain KY9611 and its *ltsA* mutant KY9714. Selective staining of mycolic acid-containing layers and peptidoglycan were performed using fluorescent probes, DHPE and Van-FL, respectively. Fluorescence images are shown in pseudocolor. Differential interference contrast (DIC) microscopic images are also presented. Arrows represent the cell division sites of *C. glutamicum* cells. Two sets of typical images for each strain are shown. A bar represents 2 μm.

We previously developed a differential staining method for the detection of cell surface layers with fluorescence dyes, including the cytoplasmic membrane, peptidoglycan layer and mycolic acid-containing layer in *C. glutamicum* [26]. Cell-surface structures in the *ltsA* mutant KY9714 were examined using this method. Mutation in the *ltsA* locus of the KY9714 strain is a substitution of the codon for the 132nd tryptophan residue to the amber stop codon. As depicted in Figure 3, the cell surface was uniformly covered with the mycolic acid-containing layer in the wild-type strain KY9611. On the other hand, in the *ltsA* mutant strain KY9714, the mycolic acid-containing layer was not detected at the nascent cell division planes in the dividing cells, which are seen as a V-shaped arrangement of cells, while a peptidoglycan layer was formed. Furthermore, formation of the peptidoglycan and mycolic acid-containing layers in other *ltsA* mutants, YK06 and YK39, carrying *ltsA9706* and *ltsA11939* alleles, respectively, was examined. As shown in Supplementary Figure S2, the mycolic acid-containing layer at the cell division planes in the dividing cells was not observed in both YK06 and YK39 strains, similar to the KY9714 strain. Almost all V-shaped snapping cells showed a defect in mycolic acid-containing layer at the division planes in these *ltsA* mutant strains. These results suggest that the *ltsA* mutations somehow affect the formation of the mycolic acid-containing layer at the cell division planes.

**Figure 4:**
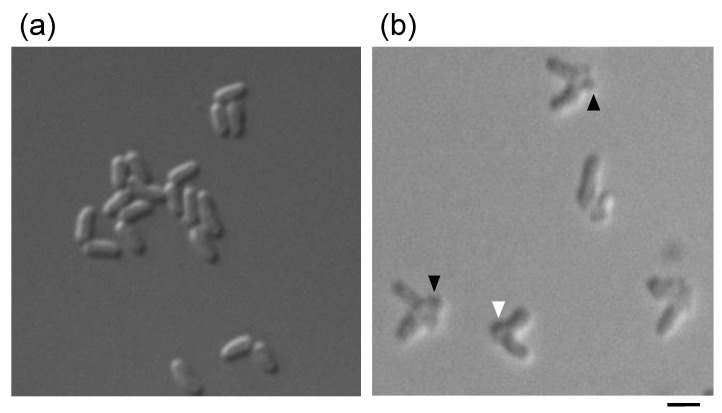
Bulge formation at cell division sites in the *ltsA* mutant upon lysozyme treatment. Differential interference contrast (DIC) microscopic images of the wild-type strain KY9611 (**a**) and its *ltsA* mutant strain KY9714 (**b**) are shown. Black arrow heads represent the cells with two bulges and the white arrowhead refers to a cell with one bulge. A bar represents 2 μm.

To evaluate the defect in mycolic acid-containing layer formation in the *ltsA* mutant, we carefully observed the lysis process of the *ltsA* mutant cells upon lysozyme treatment. The *ltsA* mutant cells were treated with lysozyme on a glass slide coated with the soft agar medium containing 20% sucrose. In the presence of 20% sucrose, it was expected that cells would not burst immediately but form a bulge at the site where the cell wall is damaged. As shown in Figure 4, bulge formation was detected at the nascent cell division sites and then a cell burst emanated from the bulge in the *ltsA* mutant cells. In contrast, the wild-type cells did not exhibit bulge formation or cell burst. This result indicates that the peptidoglycan layer at the nascent division plane, where mycolic acid-containing layer formation was defective in the *ltsA* mutant cells, was initially attacked by lysozymes. This means that the peptidoglycan layer at the division planes is exposed to the extracellular environment. Our results suggest that the lack of amidation of DAP residues in the peptidoglycan owing to the *ltsA* mutation affects the formation of a mycolic acid-containing layer at the cell division sites.

## 4. Discussion

*C. glutamicum* is highly tolerant to the lytic enzyme lysozyme despite that it is a Gram-positive bacterium. It is widely believed that the outermost layer mainly composed of mycolic acids, the mycolic acid-containing layer, is responsible for this tolerance. We previously isolated the *ltsA* mutants of *C. glutamicum,* which showed increased sensitivity to lysozyme [20]. Homology analysis showed that the LtsA protein belongs to a family of *purF*-type glutamine-dependent amidotransferases, which catalyze amide nitrogen transfer from glutamine to various acceptor substrates. Recently, it was reported that the LtsA protein of *C. glutamicum* catalyzes the amidation of DAP residues of the lipid intermediate molecules during peptidoglycan biosynthesis [25]. Consequently, DAP amidation during the synthesis of the peptidoglycan layer is deficient in *ltsA* mutant cells.

In this study, we first investigated the intracellular localization of the LtsA protein by GFP-fusion analysis and indirect immunofluorescence microscopy with anti-LtsA antibodies. We detected that peripheral localization of LtsA protein and septum localization in *C. glutamicum* cells, which is a characteristic of membrane-associated proteins (Figure 1). As the LtsA protein does not have typical transmembrane sequences or signal sequences for secretion, it seems that the LtsA protein is associated with the inner surface of the cytoplasmic membrane. As reported by Levefaudes et al., the acceptor substrates of LtsA are the lipid intermediates of peptidoglycan biosynthesis, namely lipid intermediates I and II [25]. Therefore, it is likely that the LtsA protein is associated with the inner surface of the cytoplasmic membrane via these lipid intermediates. This idea is further supported by the observation of intracellular localization of the mutant LtsA proteins. The LtsA9706 mutant protein, which has the G80D amino acid substitution in the N-terminal domain, featured similar peripheral localization to the wild-type LtsA. On the other hand, LtsA11939, which has the P559S amino acid substitution in the C-terminal domain, and the LtsAΔC, which is truncated in its C-terminal portion, did not exhibit such peripheral localization. The N-terminal GAT domain in the *purF*-type amidotransferase family is highly conserved and involved in the amido transfer reaction [22,23,24,30], while the C-terminal domain is not conserved in this family and thought to be responsible for the recognition of its own specific acceptor substrates (i.e., DAP residues in the lipid intermediates). Of note, the 559th proline is conserved among a set of LtsA homologs, such as the LtsA from *Mycobacterium tuberculosis* and *Rhodococcus erythropolis*, AsnB and AsnO from *Bacillus subtilis* and AsnB1 of *Lactobacillus plantarum*, but not among AsnB of *E. coli* or Asn1 from *Schizosaccharomyces pombe* (data not shown). The *ltsA* mutant of *R. erythropolis* showed lysozyme sensitivity [31]. Moreover, AsnB of *B. subtilis* and AsnB1 of *L. plantarum* amidate DAP residues in peptidoglycan [32,33]. These reported function of LtsA homologs having the conserved proline residue in C-terminal domain, together with our results, indicate that the C-terminal domain of LtsA is required for its function, which is presumed as recognition of lipid intermediates. Therefore, the LtsA11939 and LtsAΔC mutant proteins would lose the ability to recognize the lipid intermediates, and therefore could not localize on the inner surface of the cytoplasmic membrane. As *C. glutamicum* cells perform polar growth, peptidoglycan is actively synthesized at cell division sites and cell poles rather than side wall [34]. Therefore, the lipid intermediates of peptidoglycan synthesis are expected to accumulate at cell division sites and cell poles. The LtsA protein consequently localizes at cell division sites and cell poles by recognizing lipid intermediates with its C-terminal domain. It is reported that disruption of *L. plantarum asnB1* gene caused filamentation of cells and formation of chained cells, a characteristic of cell division mutant, suggesting that AsnB1, a homolog of LtsA, functions at cell division sites [33].

We then examined the effect of the *ltsA* mutation on the formation of cell surface structures by differential staining with fluorescent dyes. As reported previously, the mycolic acid-containing layer can be visualized by staining with phospholipid analog DHPE labeled with fluorescent dyes, while the peptidoglycan layer can be visualized by staining with Van-FL [26]. As shown in Figure 3, the wild-type cells were uniformly covered with a mycolic acid-containing layer. On the other hand, the *ltsA* mutant strain KY9714 had a defect in part of the mycolic acid-containing layer. In addition, the mycolic acid-containing layer at the nascent division plane in the dividing cells disappeared in the *ltsA* mutant cells. Instead, the peptidoglycan layer was exposed to the extracellular environment.

The formation of a mycolic acid-containing layer, which is detected by staining with phospholipid analog DHPE, is defective at the cell division site in the *ltsA* mutant, while formation of the peptidoglycan layer, which is detected by staining with Van-FL, was normal (Figure 3 and Supplementary Figure S2). Similar defect in mycolic acid-containing layer formation was observed in *C. glutamicum* cells treated with ethambutol as reported previously [26]. Mycolic acid-containing layer is composed of mycoloyl trehalose (outer leaflet of the mycolic acid-containing layer) and mycolic acid covalently bound to arabinogalactan (inner leaflet), which is linked to peptidoglycan [19,35,36]. Ethambutol affects mycolic acid-containing layer formation by inhibiting arabinogalactan synthesis [37,38]. Considering all these, it is suggested that arabinogalactan layer formation is affected in the *ltsA* mutant in the absence of amidation of DAP residues in peptidoglycan. In fact, it was shown that the *ltsA* mutant had decreased arabinogalactan bound to peptidoglycan [25]. As a result, the formation of a mycolic acid-containing layer is affected in the *ltsA* mutant.

When *C. glutamicum* cells have defects in the formation of a mycolic acid-containing layer, it is thought that the peptidoglycan layer becomes exposed, inducing the lysozyme-sensitive phenotype of *C. glutamicum*. Indeed, ethambutol-treated *C. glutamicum* cells exhibited lysozyme sensitivity (data not shown). As shown in Figure 4, bulge formation at cell division sites was observed in the *ltsA* mutant of *C. glutamicum* upon lysozyme treatment. Moreover, cells with one bulge and two bulges at the division site were observed. It seems that cells with one bulge exhibiting lysis event at the defective cell division sites and cells with two bulges are produced when septum formation is completed, but the two daughter cells are not separated. Our results indicate that in the *ltsA* mutant of *C. glutamicum*, formation of a peptidoglycan layer at the cell division site and cell separation can be completed, but the mycolic acid-containing layer at the septum is not formed.

Upon ethambutol treatment, the formation of mycolic acid-containing layer was affected both at cell division sites and cell poles [26], while it was affected only at cell division sites in the *ltsA* mutant cells (Figure 3 and Supplementary Figure S2). This is probably because the inhibition of mycolic acid-containing layer formation is transient in the *ltsA* mutant. That is, the formation of mycolic acid-containing layer is just delayed in the *ltsA* mutant cells. It was shown that mycolic acid-containing layers are highly fluid [39]. The lacking area is expected to be covered with mycolic acid by the diffusion due to the fluidity of mycolic acid layers. If this is also possible in the *ltsA* mutant cells, the surface at cell poles, that is old division sites, is covered with mycolic acid during a cell cycle. Since it has been proposed that peptidoglycan layers form a diffusion barrier for fluid mycolic acid layers, *ltsA* mutations may affect the fluidity of mycolic acid-containing layers, which causes delayed formation of mycolic acid-containing layers at the nascent division planes.

As we reported previously, certain *C. glutamicum ltsA* mutants showed temperature-sensitive growth [20,21]. It has been reported that defective mutants in terms of mycolyltransferases involved in mycolic acid-containing layer formation in *C. glutamicum* exhibit temperature sensitivity [40]. In addition, a deletion mutant of *pks13* gene encoding polyketide synthase also shows deficient mycolic acid production and temperature-sensitive growth [41]. Taken together, temperature-sensitive growth with respect to the *ltsA* mutation might be a concomitant phenotype based on the defect in mycolic acid-containing layer formation.

Emergence of multidrug-resistant *Mycobacterium tuberculosis* that is closely related with *C. glutamicum* has been a serious problem in chemotherapy. Since the mycolic acid-containing layer functions as a barrier against host defense systems, LtsA homologs of this pathogenic species could be a target of antituberculosis drugs. Such drugs targeting LtsA would not generate resistant strains because the LtsA is not essential for cell growth. In the presence of such drugs, *M. tuberculosis* cells may be eliminated by host defense systems due to a defect of the mycolic acid-containing layer. It is noteworthy that LtsA homologue of *Mycobacterium smegmatis* found as a gene conferring drug resistance [42], implying such inhibitors can be utilized in combination with other drugs to increase the susceptibility.

## 5. Conclusions

In conclusion, we found that the LtsA protein peripherally localized in *C. glutamicum* cells and that its C-terminal region was required for its localization. During peripheral localization of the LtsA protein in *C. glutamicum*, the LtsA protein probably recognizes the lipid intermediates of peptidoglycan biosynthesis containing DAP and the C-terminal region might be necessary for this recognition. Staining of the cell surface layer revealed that the peptidoglycan layer is exposed to the exterior of the cells in the *ltsA* mutants, which results in its lysozyme sensitivity.

## Data Availability

The data presented in this study are available in this article and supplementary materials.

## Supplementary Materials

The following are available online at https://www.mdpi.com/2076-2607/9/2/409/s1, Figure S1: Complementation of temperature sensitivity and lysozyme sensitivity of the *ltsA* mutant KY9714 by the *gfp*-fused wild-type *ltsA* gene. Figure S2: Defect in formation of mycolic-acid containing layer at cell division site in the C. *glutamicum ltsA* mutants YK06 and YK39.
