# Supplementary material for: Requirement of the LtsA Protein for Formation of the Mycolic Acid-Containing Layer on the Cell Surface of Corynebacterium glutamicum
Source: Microorganisms. 2021 Feb 16;9(2):409. doi: 10.3390/microorganisms9020409 (PMC7920481; doi:10.3390/microorganisms9020409)
Supplement: Supplementary file 1 [file microorganisms-09-00409-s001.pdf]

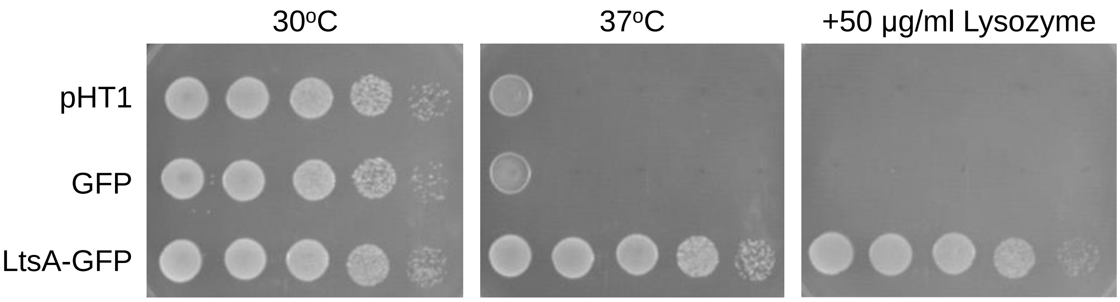

Supplementary Figure S1. Complementation of temperature sensitivity and lysozyme sensitivity of the *ltsA* mutant KY9714 by the *gfp*-fused wild-type *ltsA* gene. Serially-diluted cultures of the KY9714 transformed with the empty vector (pHT1), plasmid expressing GFP alone (GFP) or plasmid expressing LtsA-GFP (LtsA-GFP) were spotted on L plates or L plate containing 50 µg/ml lysozyme and the plates were incubated at 30 (left and right panels) and 37°C (center) for 1 day.

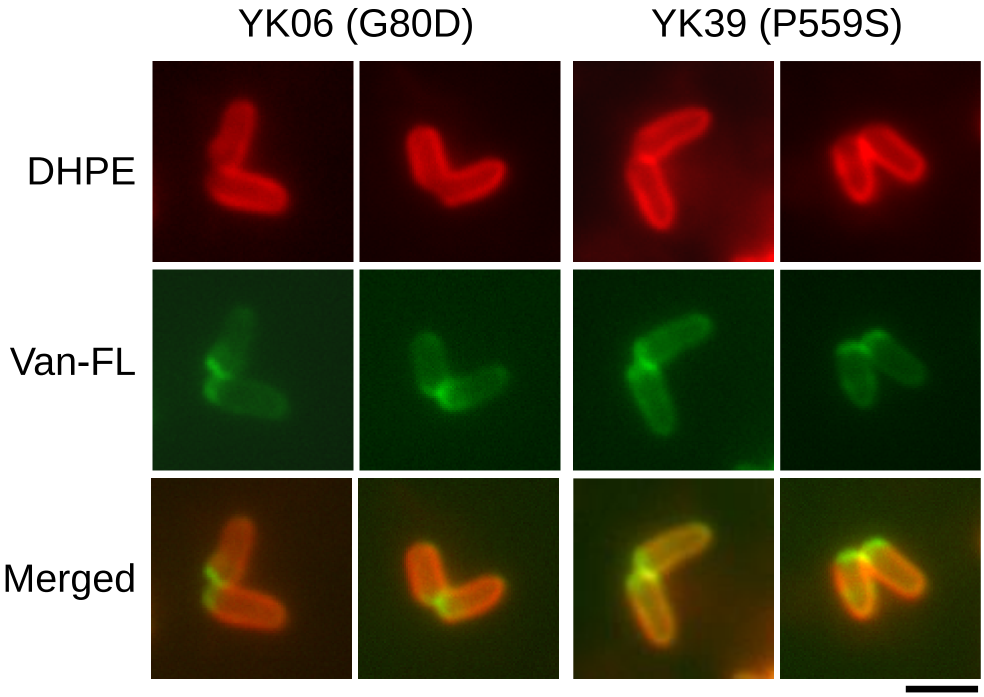

Supplementary Figure S2. Defect in formation of mycolic-acid containing layer at cell division site in the *C. glutamicum ltsA* mutants YK06 and YK39. Selective staining of peptidoglycan and mycolic acid-containing layers were performed by using fluorescent probes Van-FL and DHPE, respectively. Fluorescent images are shown in pseudocolor. A bar represents 2 μm.
